# p63 and smooth muscle actin expression in low‐grade spiradenocarcinomas in a case of CYLD cutaneous syndrome

**DOI:** 10.1111/cup.13311

**Published:** 2018-07-19

**Authors:** Aoisha Hoyle, Kerry Davies, Neil Rajan, Lucy Melly

**Affiliations:** ^1^ Department of Pathology Southern General Hospital Glasgow UK; ^2^ Institute of Genetic Medicine Newcastle University Newcastle upon Tyne UK

**Keywords:** CYLD, cylindroma, p63, SMA, spiradenocarcinoma

## Abstract

Low‐grade spiradenocarcinoma is a rare skin tumor, with fewer than six reported cases, arising in the context of CYLD cutaneous syndrome (CCS; syn: Brooke‐Spiegler syndrome [BSS]). We report two independent cases of spiradenocarcinoma arising in a 50‐year‐old man with CCS. The tumors grew rapidly, prompting clinical excision. The histologic features in our cases corresponded to the salivary gland type basal cell adenocarcinoma‐like pattern, low grade (BCAC‐LG), that has been previously recognized as a recurrent finding in CCS. We performed genetic testing of the patient and found a novel mutation in *CYLD.* Recognition of low‐grade spiradenocarcinoma can be difficult for the pathologist as the features can be subtle, especially in those cases where the transition from benign to malignant tumor is gradual. We examined p63 and smooth muscle actin (SMA) expression patterns in BCAC‐LG and compared it with the expression pattern in the precursor spiradenoma. Our report provides information on two rare tumors in the context of CCS and suggests that the pattern of p63 and SMA staining may aid pathological detection of the BCAC‐LG subtype of spiradenocarcinoma.

## 1 INTRODUCTION

Low‐grade spiradenocarcinoma is a rare skin tumor that may arise in CYLD cutaneous syndrome (CCS). Recently, *CYLD* loss as a genetic driver in inherited cylindromas and spiradenomas, associated with increased MYB signaling, has been recognized in CCS.^1^ In contrast, sporadic cutaneous cylindromas recurrently harbor *MYB‐NFIB* fusion transcripts^2^ that result in MYB overexpression. The convergence on MYB signaling in inherited and sporadic tumors suggests that the biology of sporadic spiradenocarcinoma may be informed by the study of CCS patients. We report a case of two spiradenocarcinomas arising in a 50‐year‐old man with CCS. The histologic features in our case corresponded to the salivary gland type basal cell adenocarcinoma‐like pattern, low grade (BCAC‐LG), a type previously recognized as predominating in CCS. Recognition of BCAC‐LG spiradenocarcinoma can be difficult in routine practice as the features can be subtle and easily missed, especially in those cases where the transition from benign to malignant tumor is gradual. We examined the expression pattern of p63 and smooth muscle actin (SMA) in our two cases of BCAC‐LG spiradenocarcinoma and suggest that these immunohistochemical markers may aid in the distinction of BCAC‐LG spiradenocarcinoma from spiradenoma in CCS.

## 2 CASE REPORT

**Figure 1:**
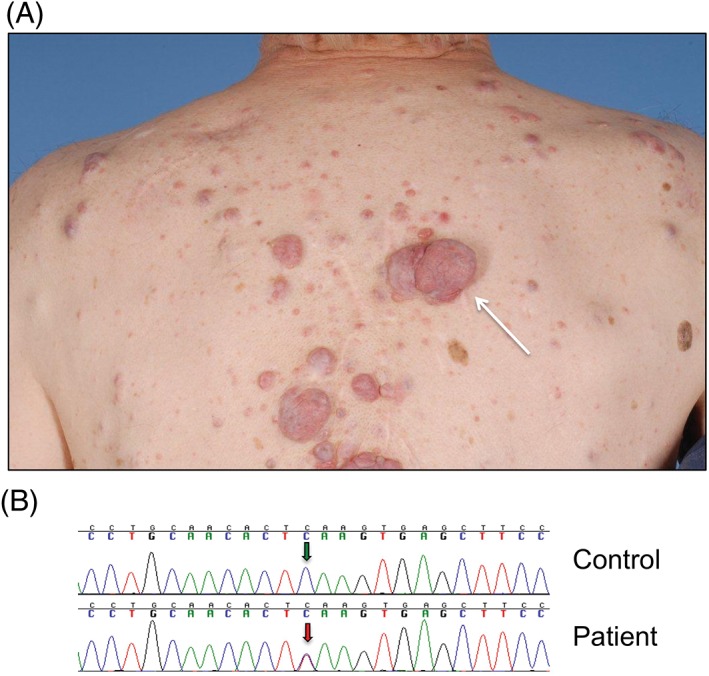
A, Clinical image of patient with malignant spiradenocarcinoma arrowed (white arrow). Note the intact epidermis and the lack of blue regions within the nodule. B, A novel heterozygous germline mutation in *CYLD*

A 50‐year‐old male presented with a rapidly enlarging lesion on his back. He had multiple skin lesions affecting most of his upper body, arms, and face (Figure 1A). The patient had first noticed skin tumors around the age of 18 years, and had multiple lesions removed, which were confirmed as cylindromas and spiradenomas. He was severely affected with multiple scalp tumors, such that he underwent total scalp excision with skin grafting. He had affected relatives, and sequencing of the *CYLD* gene in peripheral lymphocyte DNA revealed a novel pathogenic heterozygous truncating mutation (c.2476C>T; p.Gln823*) (Figure 1B), consistent with a diagnosis of CCS. The patient underwent surgical excision of the lesion on the back, which showed low‐grade spiradenocarcinoma. Two years after this a further enlarging tumor was excised from his suprapubic skin and confirmed to be a primary spiradenocarcinoma. Five years since his first spiradenocarcinoma, neither lesion has recurred.

**Figure 2:**
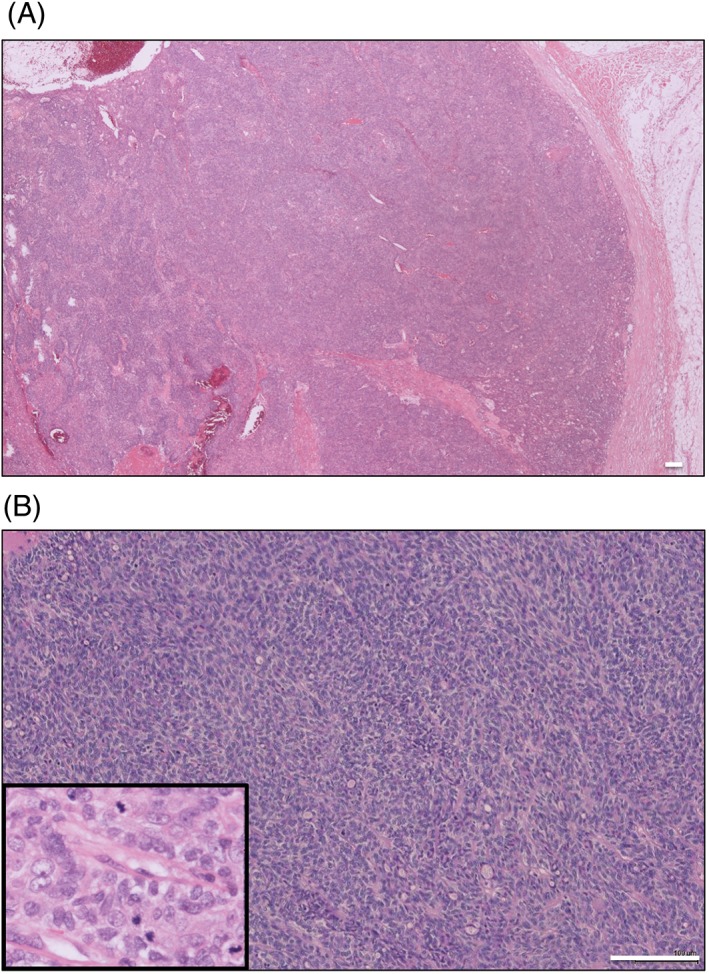
H+E (A, ×20) Low‐grade spiradenocarcinoma showing nodular growth pattern and lack of infiltrative growth. (B, ×200) showing the lack of a dual cell population. Mitotic figures within the malignant component are shown in the inset (×400). Scale bars indicate 100 μm

Histopathology of the tumor from his back showed an ulcerated spiradenocarcinoma arising in a spiradenoma. The spiradenocarcinoma was characterized by increased cellularity and absence of the dual cell population seen in spiradenoma. The neoplastic cells were arranged in nodules and had minimal cytoplasm, and some showed a slightly spindled morphology (Figure 2A,B). The ductal structures (highlighted by carcinoma embryonic artigen staining; data not shown) appeared compressed and pushed to the periphery. There was loss of the diffuse infiltrate of small lymphocytes. Within the neoplastic nodules, the cells showed increased mitotic activity (15/10 hpf). The spiradenoma in comparison was characterized by a dual population of cells arranged in trabeculae. The cells were a mixture of small basaloid cells with small dark nuclei, and a second cell type with a larger more irregular vesicular nucleus and more cytoplasm. Mitotic activity was very low in the benign component (1/10 hpf).

**Figure 3:**
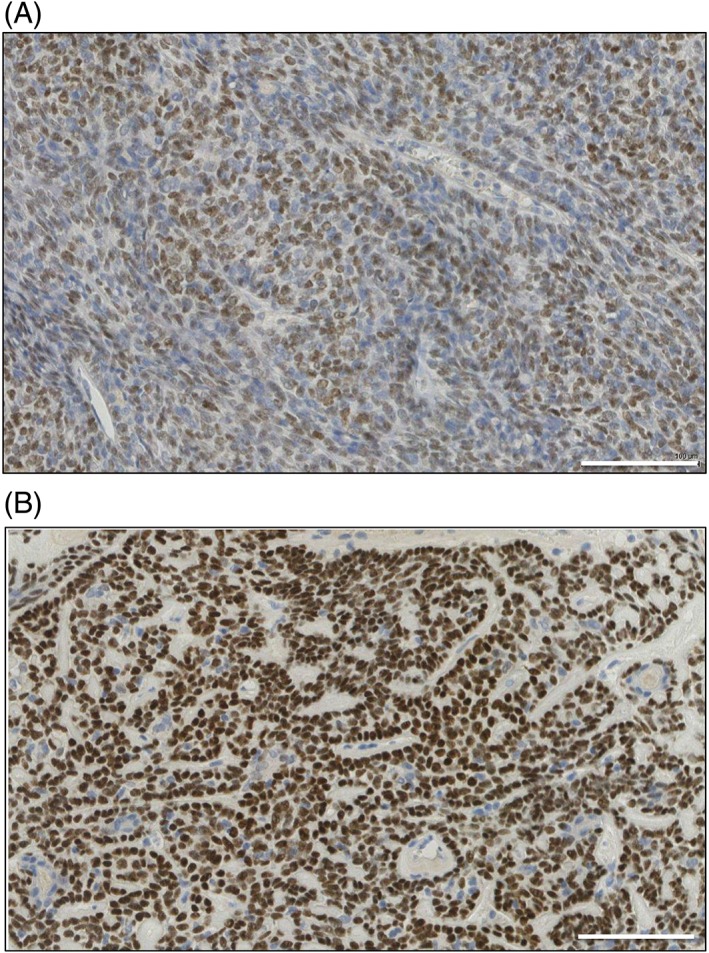
p63 (A, ×200) staining in low‐grade spiradenocarcinoma of the trunk, showing uniform weak p63 staining, with some p63 negative cells. (B, ×200) Benign precursor spiradenoma showing a majority of strong nuclear p63 staining and occasional weak and negative p63 staining cells. Scale bars indicate 100 μm

**Figure 4:**
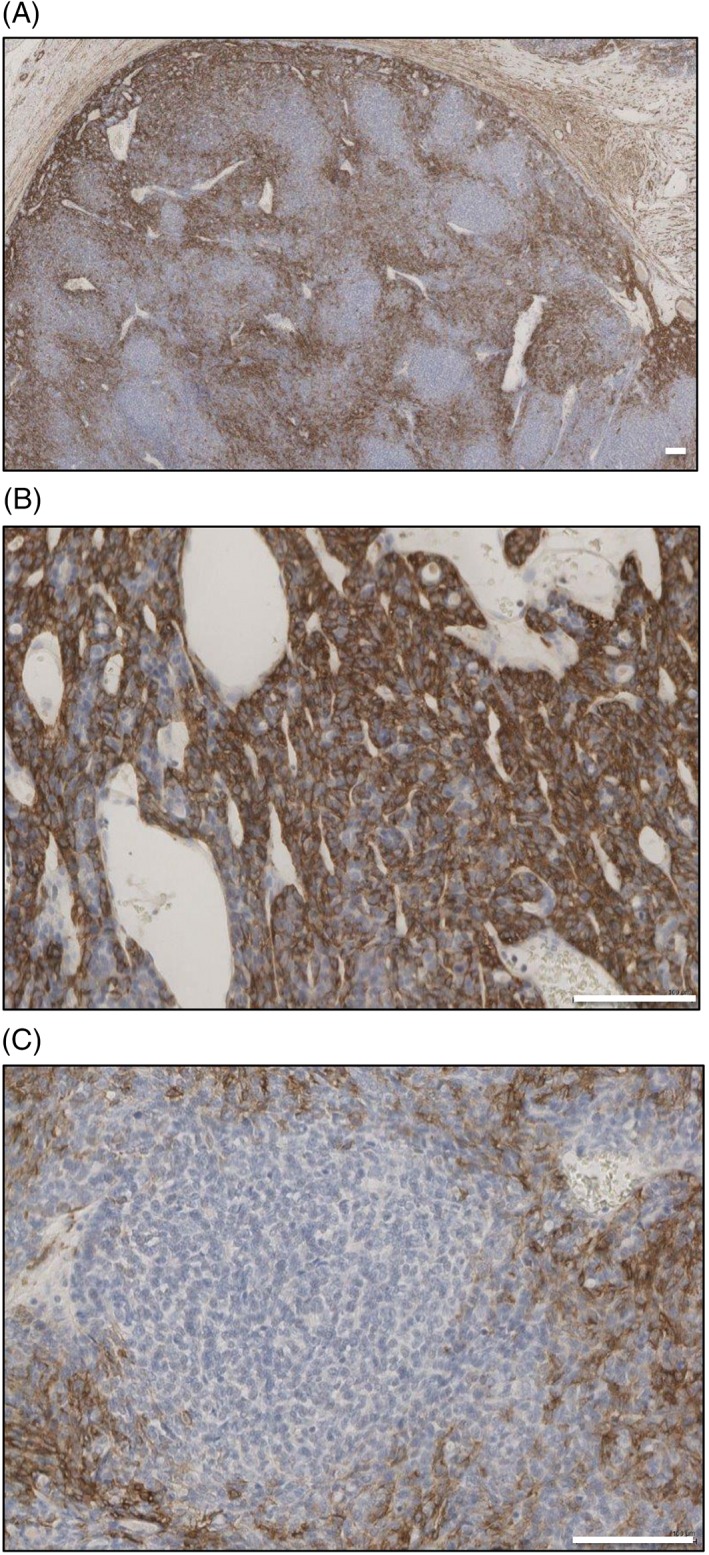
Smooth muscle actin (SMA) (A, ×40) staining showing the nodules of SMA negative cells in spiradenocarcinoma. (B, ×200) SMA staining pattern in benign spiradenoma component and (C, ×200) malignant spiradenocarcinoma. Scale bars indicate 100 μm

Immunohistochemistry for p63 and SMA was performed on spiradenoma and spiradenocarcinoma. p63 showed strongly positive cells closely associated with occasional weak p63 staining cells in spiradenoma and showed uniform but weak p63 staining in spiradenocarcinoma (Figure 3A,B). SMA showed a mixture of SMA positive cells and SMA negative cells diffusely throughout the lesion in spiradenoma, and nodules of SMA negative cells with SMA positive cells compressed at the periphery in spiradenocarcinoma (Figure 4A‐C). These nodules were most easily recognized on low power magnification (Figure 4A). Similar features were seen in the spiradenocarcinoma arising in the suprapubic skin.

## 3 DISCUSSION

Spiradenocarcinoma is a rare dermal appendage tumor, with approximately 100 cases reported in the literature. There is no sex predominance, and the age range is 30 to 92 years, with an average age of 59 years.^3^ Kazakov et al reported 24 cases of malignant neoplasms arising in pre‐existing spiradenoma, cylindroma, and spiradenocylindroma and importantly included five cases arising in CCS.^4^ Kazakov et al suggested classifying spiradenocarcinoma into four histological subtypes, namely: salivary gland type basal cell adenocarcinoma‐like pattern, low grade (BCAC‐LG); salivary gland type basal cell adenocarcinoma‐like pattern, high grade (BCAC‐HG); invasive adenocarcinoma not otherwise specified (IAC‐NOS); sarcomatoid (metaplastic) carcinoma. The BCAC‐LG and BCAC‐HG patterns relate to those described in Granter's description^5^ where in the low‐grade spiradenocarcinomas, the transition between malignant and pre‐existing benign cells is gradual, whereas in the high‐grade spiradenocarcinomas, it is abrupt. Both patterns showed loss of the dual cell population and lymphoid infiltrate. Of the five malignant tumors arising in CCS, three had BCAC‐LG, and two BCAC‐HG.^4^ Recently a review of 19 cases of BCAC‐LG (1 case in the context of CCS), the pattern most often reported in CCS, showed a relatively indolent course, characterized by local recurrence.^6^ We add to the six reported cases of BCAC‐LG arising in the context of CCS,^4^, ^6^ with two additional cases arising in a genotyped patient with a novel *CYLD* mutation.

CCS is an inherited autosomal dominant disease with variable expression and incomplete penetrance.^7^ It results in the growth of multiple adnexal neoplasms from the follicular‐sebaceous‐apocrine unit especially trichoepitheliomas, cylindromas, spiradenomas, and spiradenocylindroma. The presence of multiple such tumors or a family history allows for clinical diagnosis. CCS can present in any of three patterns, namely familial cylindromatosis (FC) where the predominant tumors are scalp cylindromas, multiple familial trichoepitheliomas, where the predominant tumors are facial trichoepitheliomas, and Brooke‐Spiegler syndrome (BSS), where any combination of trichoepitheliomas, cylindromas and related spiradenomas are seen.^7^ Genetic diagnosis as performed in our case is now possible and aids broader issues such as genetic counseling of the patient's family. *CYLD* is a tumor suppressor gene that plays a regulatory role in immunity, development, inflammation, and lipid metabolism. There are more than 110 known mutations in *CYLD*, and the majority are truncating mutations resulting predicted loss of function, although splice site and missense mutations are also seen.^7^, ^8^ It is striking that two primary low‐grade spiradenocarcinoma arose in our case when the patient was 50 and 52 years of age. Taken together with the cases reported previously,^4^ low‐grade spiradenocarcinoma appears to develop in older patients with CCS, with the range of reported ages at presentation of seven cases ranging from 43 to 72 years.

We suggest that p63 and SMA immunohistochemistry may assist histologic detection of the BCAC‐LG subtype of spiradenocarcinoma. Further studies are needed to fully assess the usefulness of p63 and SMA in this context. Brenn et al highlight the consistent reduction or loss of MYB expression as a useful marker of low‐grade spiradenocarcinoma when compared to the benign component it has arisen from.^6^ p53 expression and Ki‐67 by contrast were of limited use in discriminating malignant components due to variability of expression across the cases assessed. p63 expression has been described in a single case of sporadic BCAC‐LG spiradenocarcinoma.^9^ The authors showed a difference in staining between benign and malignant components, compatible with our findings. We provide additional data on the pattern and intensity of p63 and SMA expression in CCS, suggesting that these markers may aid the histopathologic recognition of BCAC‐LG spiradenocarcinoma. Taken together with the morphological features seen in BCAC‐LG type spiradenocarcinoma, these immunohistochemical markers may aid diagnosis; however, further studies in a large series are important to confirm this impression.

In summary, we present a patient with two cases of spiradenocarcinoma, arising in a setting of CCS. Subclassification of subtypes appears to be important, as it has been suggested that the histology influences prognosis. Our follow‐up data is supportive of the relative indolent clinical course BCAC‐LG type spiradenocarcinoma in CCS has been noted to have, and this may influence the clinical management of these rare patients.
